# From Modes to Movement in the Behavior of *Caenorhabditis elegans*

**DOI:** 10.1371/journal.pone.0013914

**Published:** 2010-11-16

**Authors:** Greg J. Stephens, Bethany Johnson-Kerner, William Bialek, William S. Ryu

**Affiliations:** 1 Joseph Henry Laboratories of Physics and Lewis–Sigler Institute for Integrative Genomics, Princeton University, Princeton, New Jersey, United States of America; 2 Department of Physics, Banting and Best Department of Medical Research, University of Toronto, Toronto, Canada; Lund University, Sweden

## Abstract

Organisms move through the world by changing their shape, and here we explore the mapping from shape space to movements in the nematode *Caenorhabditis elegans* as it crawls on an agar plate. We characterize the statistics of the trajectories through the correlation functions of the orientation angular velocity, orientation angle and the mean-squared displacement, and we find that the loss of orientational memory has significant contributions from both abrupt, large amplitude turning events and the continuous dynamics between these events. Further, we discover long-time persistence of orientational memory in the intervals between abrupt turns. Building on recent work demonstrating that *C. elegans* movements are restricted to a low-dimensional shape space, we construct a map from the dynamics in this shape space to the trajectory of the worm along the agar. We use this connection to illustrate that changes in the continuous dynamics reveal subtle differences in movement strategy that occur among mutants defective in two classes of dopamine receptors.

## Introduction

From the swimming motions of *E. coli* [1] to the mobility of human populations [2], the way in which organisms move through the world profoundly influences their experience. Ultimately, these strategies of movement change the chances for survival and reproduction, and thus are subject to natural selection. Typically, ecological or evolutionary studies of movement focus on trajectories measured through course-grained variables such as the center of mass [3]. However, to an organism, movement is not translation or rotation of its body relative to an external coordinate system, but rather transformations of shape as measured in its own intrinsic coordinates. Analyzing together both the internal states and external motions offers deeper insight into the study of movement strategies.

In general, the connection between transformations in shape space and movement through the world is complicated. There is a long tradition of work which tries to make this connection through analytic approximations of the equations describing the mechanics of the organism's interaction with the outside world. This approach is perhaps best developed for swimming and flying organisms [4], [5], and there are particularly elegant results in the limit of swimming at low Reynolds number [6]–[8]. All of these methods depend on some small parameter in the physical interaction between the organism and its environment. A very different possibility for simplifying the relation between shapes and movement arises if the space of shapes itself is limited. In several systems, the potentially high dimensional space of shapes or movements is not sampled uniformly under natural conditions, so that one can recognize a lower dimensional manifold that fully describes the system [9]–[12]. In these cases it is possible to ask empirically how motions on this low dimensional manifold map into movements relative to the outside world.

The motion of the nematode *Caenorhabditis elegans* provides an example of this dimensionality reduction. In previous work, we found that the shapes taken on by the worm's body are well-approximated by a four dimensional space spanned by elementary shapes or ‘eigenworms’ [13]. Here we connect the dynamics in this low dimensional space of shapes to the trajectories of worms as they crawl on an agar plate, the conventional experimental setup for studying worm behavior [14]–[17]. In the process, we offer a new analysis of the trajectories themselves, and show how the intrinsic shape dynamics gives us a more comprehensive tool for the analysis of mutant locomotory behavior.

## Results

### Center-of-mass trajectories

**Figure 1:**
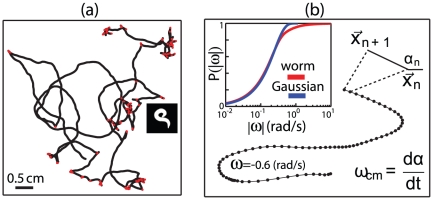
Center-of-mass trajectories. (a) A typical center-of-mass trajectory on the agar plate. The path includes both abrupt orientation changes (red) in which the worm shape is deeply bent (inset), and continuously curving segments. (b) Definition of the local orientation angle 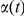 and the local orientation velocity 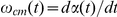. The inset shows the cumulative distribution of orientation velocities (red) contrasted with a Gaussian distribution that has the same variance (blue). The center-of-mass time series 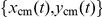 was filtered with a third-order polynomial in a running window 11 frames in length and 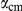 and 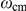 were built using this filter.

To understand how changes in the worm's shape determines its motion, we first characterize the motion itself. In Fig 1a, we show an example of the worm's trajectory, defined by the centroid of the black and white body images. The worm's trajectory consists of gently curving segments, interrupted by sharp turns or reversals. These discrete reorientation events are characterized by curved body shapes, such as turns caused by body bends or turns after reversal events, known as 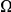
–turns, because the worm's resemblance to the Greek letter 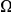. It is tempting to think of these trajectories as being approximately like those of *E. coli*, consisting of long, relatively straight runs punctuated by tumbles, which randomly reorient the cell [18]. Indeed, variations of this model in which trajectories are segmented into discrete ‘runs’ have long been used to study the movements of a wide variety of organisms [19], [20] including *C. elegans* [14], [21]. However we will see the limitations of this “run and tumble” model by closer statistical analysis of the entire trajectory.

Trajectories are characterized by the speed of motion, 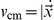, the local tangent angle, 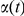, and the local orientation velocity 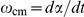 at each moment in time (Fig 1b). The standard deviation of the curvature is 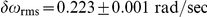, but the distribution of angles 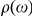 has long tails, as shown by comparing the cumulative distribution 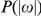 to a Gaussian distribution with the same variance (inset to Fig 1b). Excursions to the large amplitude tails correspond to abrupt reorientation events and are colored red in Fig 1a.

**Figure 2:**
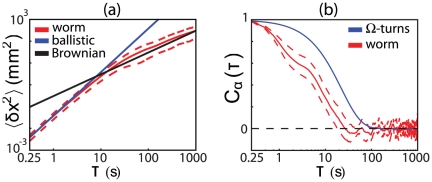
Trajectory correlations. (a) Mean–square displacement as a function of time along the trajectory [Eq (1)]. At short times we see ballistic (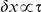) behavior, crossing over to diffusion [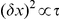] at long times. (b) The orientation angle correlation function [Eq (2)] for worm motion (red), contrasted with a simple “run and tumble” model (blue) in which reorientation events occur randomly with rate 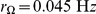, corresponding to the rate of reorientation events [14], [21].

If worms lose orientational memory then their movements will look diffusive at long times. One signature of such behavior is contained in the mean–square distance between two points on the trajectory,
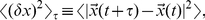
(1)which for diffusion should grow linearly with the time 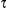. In Fig 2a, we see that this is what happens for times longer than 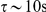. At shorter times, the mean–square displacement grows as the square of the time difference, which corresponds to ballistic motion at a fixed velocity. This suggests that the time scale for worms to lose directional memory is 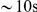, and this can be seen directly from the correlation function for the local tangent angle,
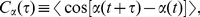
(2)as shown in Fig 2b.

A sequence of uncorrelated runs and tumbles translates into a mathematical model in which the orientation angle 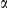 is, from time to time, completely randomized by discrete events. For *E. coli* these events are the tumbles, and it is natural to think that for *C. elegans* these events are the reorientation events. If turns occur randomly at rate 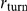, and generate completely new, uncorrelated directions of movement, then the correlation function for direction will decay as
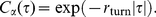
(3)But since 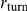 typically is less than two per minute [14], [21], reorientation events alone can't explain the shorter time of decay of the directional correlations, as seen quantitatively in Fig 1d. Thus, changes in the orientation angle 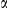 occurring in between reorientation events must play a key role.

**Figure 3:**
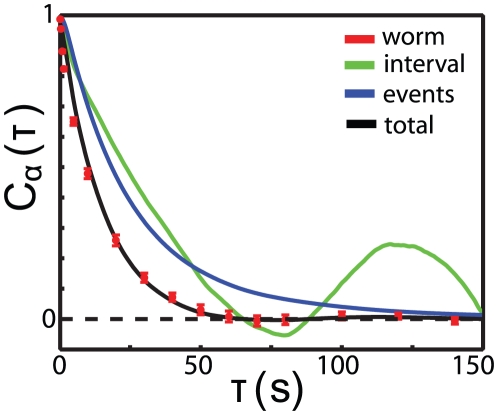
Components of orientation correlation. The loss of orientation memory is captured by independent contributions from abrupt reorientation events and continuous dynamics between these events. The orientation correlation function 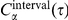 computed by averaging over all delays within the same interval is shown in green. The probability 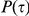 that two times separated by 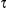 are within the same interval is shown in blue. The predicted orientation correlation function from Eq 4 is shown in black, compared with the data, shown as red points with standard errors of the mean.

We can disentangle the contributions of continuous and discrete reorientation by looking at the orientation correlation of the trajectory between reorientation events. As in previous work [21] we identify reorientation events as moments when 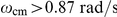. The correlation of the angles at the beginning and the end of the reorientation event, 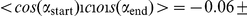
0.01, demonstrating that these turns effectively randomize the direction. We compute the orientation correlation function 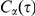 within the intervals, and the result, 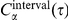, is shown as the green curve of Fig. 3. Removing the intervening abrupt reorientation events reveals a non-exponential, non-monotonic angular correlation, which is a marked departure from ordinary orientational diffusion.

If the continuous dynamics within intervals are independent of reorientation events then the angular correlation function is the product of two terms
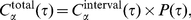
(4)where 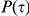 is probability that two points separated by time 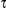 come from the same interval. To quantify the contribution of continuous dynamics to the overall loss of orientation memory, we use Eq 4 to compute 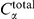 and we compare this with the measured angular correlation. Figure 3 shows that this is an excellent approximation, with no adjustable parameters. Importantly, the continuous dynamics remain significant even when the threshold is lowered to 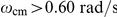, the lowest value where Eq 4 is an adequate description of orientation correlations (data not shown). These results demonstrate that the loss of total orientational memory is due to two independent processes, both of which may be controlled as part of an overall foraging strategy, and that a “run and tumble” model focusing on discrete reorientation events is an inadequate description of *C. elegans* movement.

### Modes and movements

**Figure 4:**
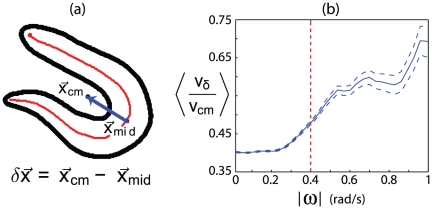
Problems of center-of-mass tracking. (a) The displacement vector 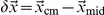. (b) Average ratio of 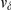 to 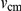 as a function of local orientation velocity. Dotted lines denote error in the mean. When the local orientation velocity, 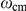 is large, the center-of-mass velocity mixes changes of the body shape with changes in the directional heading. The dotted red line denotes the threshold we use for modeling in section Modes and movements.

Worms move in the world by changing their posture and here we show that the centroid trajectories are quantitatively captured by the dynamics of body shape. Following general practice, we generated trajectories by tracking the center of mass of the worm's silhouette, 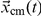, but an equally valid alternative would be to follow the midpoint of the worm as indicated in Fig 4a, generating a trajectory 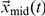. When the worm moves smoothly and doesn't change shape dramatically, both definitions of trajectory produce nearly identical results, but during those moments when the worm bends deeply, these trajectories diverge. We can quantify this divergence by measuring the difference between the center of mass and the midpoint trajectories, 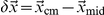 and defining the difference speed 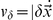. For large curvature we see this difference speed 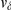 approaches the center-of-mass speed 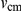 (Fig 4b), illustrating that neither measure is a good indicator of the trajectory. Guided by these observations, we partition the worm's trajectory into three regimes: (i) segments where the centroid heading is a faithful representation of trajectory orientation, 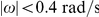, 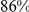 of the data; (ii) segments involving large reorientations which we collapse to point events, 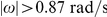, 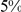 of the data; and (iii) ambiguous segments 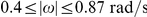, 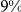 of the data. To construct the connection between shape and movement we confine our analysis to the first regime and note that our results are insensitive to small changes in the boundaries between segments.

**Figure 5:**
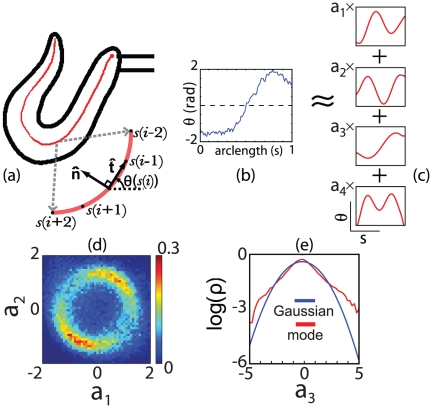
Low dimensional description of worm shape, following Ref [**13**]. (a) Each raw image of the worm is processed by passing a curve through the center of the body; the red circle marks the head. Arc length 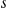 along this curve is normalized, 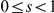, and we define the tangent 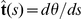 to the centerline curve. (b) We rotate all images so that 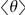 is zero and thus 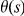 provides a description of the worm shape that is intrinsic to the worm itself. (c) We decompose each shape 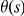 into contributions from the leading four eigenmodes, which capture 95% of the variance in shape space, and the amplitudes of fluctuation along each mode are normalized to unit variance, 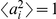. (d) Fluctuations along the first two modes correspond to an oscillation, or nearly circular orbit in this projection of the shape space. (e) Fluctuations along the third mode show strongly non–Gaussian statistics. The first two modes drive the propulsive wave along the body, the third mode makes the dominant contribution to turning, and the fourth mode (not shown) describes localized fluctuations of the head and tail.

In previous work [13] we found that the space of shapes of *C. elegans* during free locomotion is low-dimensional with four principal dimensions (eigenworms) capturing approximately 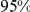 of the variance of the space of shapes. In this framework, the trajectories of worm shape are quantitatively described as a linear combination of the four eigenworms with corresponding time-varying weights, the modes 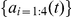. A summary of these results is shown in Fig 5 and we use these modes to construct an explicit map from the dynamics of shape into 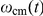.

**Figure 6:**
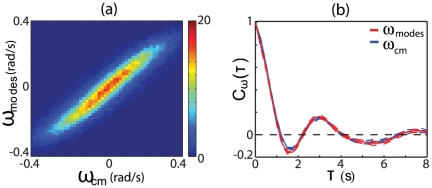
The intrinsic dynamics of worm shape capture the extrinsic motions of foraging trajectories. (a) We construct an eigenworm model of the orientation velocity (Eq 9) and show the joint distribution 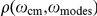. (b) The orientation velocity two-point correlation function for the eigenworm model (blue) and the center-of-mass motion (red).

In general we expect the worm's reorientation dynamics to be connected to changes in its shape, and we can construct this connection between modes and movement using the four intrinsic shape modes. The simplest model is a linear one,
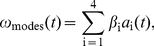
(5)and by fitting the coefficients, 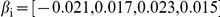 rad/s, we can capture 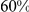 of the variance in 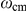. More generally we expect a nonlinear relationship, including time derivatives of the shape. Thus, we introduce twelve variables,
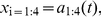
(6)

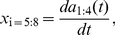
(7)

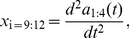
(8)and model the curvature as
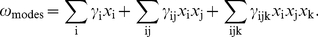
(9)This mode-based model of 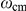 provides an excellent fit, Fig 6a, and captures more than 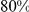 of the variance in the curvature. More importantly, we also predict the dynamics of the curvature, as shown through the correlation function
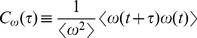
(10)in Fig 6b, and it is these dynamics that underlie the continuous reorientations shown in the green curve of Fig 3.

The curvature is dominated by contributions from the amplitude of the third mode, which is connected to bending of the worm's body [13]. We have shown previously that the instantaneous speed of the worm 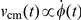, where the phase 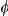 as the angle in the plane 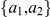 (cf Fig 5d). Taken together we can map from the modes to the speed and local curvature of the center-of-mass trajectory, so we can completely reconstruct the worm's movements from its shape as a function of time (Figure S1).

### Mutants and adaptation

As with all organisms, *C. elegans* behavior is modulated by experience. As an example, the rate of 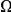
–turns decreases systematically with time away from food [14], as well as changing in response to thermal [22] and chemical [21] stimuli. It is also known that dopamine plays a significant role in these behaviors [23], [24]. Here we use the analytic tools developed above to characterize both adaptation and the behavior of dopamine mutants *dop-2 (vs105)* and *dop-3 (vs106)*.

**Figure 7:**
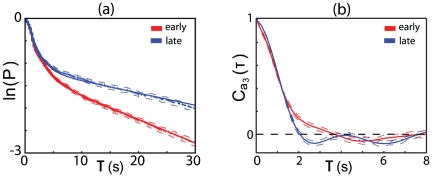
Adaptation of foraging behavior. (a) The cumulative distribution of times between reorientation events in wild–type worms. Reorientation events are defined as described in the text, and dashed lines denote bootstrap standard deviations. (b) The temporal correlation of the third eigenmode, 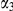, between reorientation events, for early and late times.

The distribution of times between reorientation events changes as the worms spend more time away from food. As before we identify reorientation events as moments when 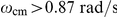. With this threshold, we can measure the cumulative distribution of inter–turn intervals, or equivalently the probability that a turn has not yet occurred after a time 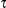, and this is shown in Fig 7a. During the course of our experiments the worms spend 40 min away from food; allowing for an initial adjustment to being placed on the the plate we divide the last 28.3 min into three equal epochs to search for adaptation to the environment. We see that the interval distributions vary systematically with time. In more detail, we see that the distribution has two components,
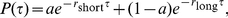
(11)and only the slower component 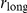 contributes to the lengthening of the times between turns. A similar results has been shown for swimming worms [25]. Repeating the analysis for the dopamine mutants, we find the interval distributions statistically identical (Table 1). The short time behavior of the distribution, summarized by 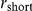 is unchanging, while the long time behavior varies across time but is different than the wild-type at late times. The dopamine mutants do not have the same suppression of reorientation events as the wild-type as shown previously [24].

**Table 1:** Properties of the inter–turn interval distribution.

	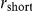 (Hz, 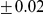 )			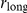 (Hz, 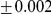 )		
genotype	early	middle	late	early	middle	late
N2	0.63	0.76	0.63	0.053	0.040	0.027
*dop-2*	0.68	0.67	0.68	0.072	0.048	0.044
*dop-3*	0.65	0.66	0.60	0.070	0.046	0.042

Data as in Fig 7 were collected also for 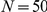 dopamine mutants, each also observed for 35 minutes, and all the data were fit to Eq 11. Results are shown for the two rates, 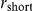 and 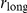, that define the time scales for turning. We note that 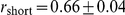 Hz across all epochs and genetic variants, and that differences in 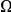
–turns between the dopamine mutants are statistically insignificant.

**Figure 8:**
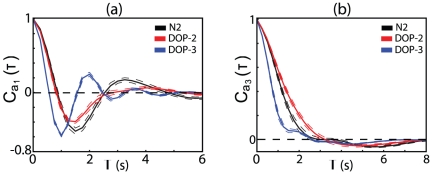
Correlation functions for the shape modes. Figures indicate correlation functions for 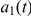 and 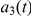, comparing the wild type 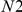 with the two mutant strains *dop-2* and *dop-3*. Shorter timescales for the *dop-3* mutants results in faster center-of-mass speed and curvier trajectories. Dashed lines denote bootstrap standard deviations.

Our analysis above shows that continuous re–orientation in between turns is a significant component of the worm's motion, and that this behavior is driven by the dynamics in shape space. Indeed, the correlation time of the bending mode 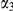 also shows adaptation between early and late times (Fig 7b), showing that worms produce straighter inter-turn trajectories at later times. Although the statistics of turning are the same for the two different mutants, Fig 8 shows that the dynamics along the different modes are in fact quite different. Along modes 1 and 2 (which form a quadrature pair), *dop-2* is similar to the wild type, but *dop-3* exhibits a faster oscillation. Recalling that this corresponds to the undulatory wave along the body [13], we predict that the *dop-3* mutant should move more quickly, and this is observed when we track the center-of-mass motions: mean speeds of the three variants are 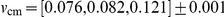 mm/s for N2, *dop-2* and *dop-3* respectively. The mode dynamics also combine though Eq 9 to produce different turning dynamics and, in particular, *dop-3* animals make longer-lasting turns which result in curvier trajectories. While these pheonotypic differences are subtle and have not been reported before, they are immediately apparent in the dynamics in shape space. Taken together we find continuous motion, including gradual re–orientation, and discrete turning behaviors are under genetic control.

## Discussion

It is tempting to think of behavior as a sequence of discrete actions, each taken in consequence of a specific decision. In the simplest cases, such as the running and tumbling of *E. coli*, we can see these discrete events reflected directly in the trajectory of the organism's center-of-mass motion [1]. In contrast, we find that, for *C. elegans*, discrete behaviors are roughly only half of the story: the exploratory trajectories of the worm get approximately equal contributions from discrete turning events and from continuous re–orientational motions in between the turns. Further, we can trace these continuous motions back to the underlying dynamics in the space of body shapes. Finally, we see that these different components of the motion are under independent genetic and adaptive control.

Quantitatively, we found that the exploratory motions of *C. elegans* are composed of two principle reorientation elements: abrupt events, including classical 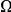
–turns, which occur infrequently, and the continuous dynamics of orientation between these events. These two processes both make significant contributions to the worm's total loss of orientational memory on the 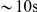 time scale. By focusing on the continuous intervals between discrete turns, however, we find that the worm's orientation can exhibit a longer term memory, lasting two minutes or more, and non-monotonic correlations, corresponding to an abundance of arcs as seen, for example, in the trajectory of Fig. 1a. The presence of these arcs as well as the differential behavior of the mutants suggests that *C. elegans* controls more aspects of its motion than the stochastic rate of abrupt events. Evidence for this sort of continuous control has also recently been observed during *C. elegans* chemotaxis [26].

Extending previous work showing that the body shapes of *C. elegans* are captured by four modes [13], we built a phenomenological model that connects the intrinsic dynamics of these modes to the speed and curvature of the worm's trajectory through the external world. This model allows us to connect the body configurations, which are what the neuromuscular system can control, to the behaviors that have adaptive value. As an example of what can be learned from this analysis, we studied the motion of two mutants, *dop-2* and *dop-3*, which contain defective receptors for the neurotransmitter dopamine, an important component in the modulation of foraging strategy. Although these mutants have nearly identical statistics when we look at their discrete turning behaviors, their continuous motions, as seen in the dynamics of fluctuations along four different shape dimensions, show substantial differences. This suggests that the goal of mapping genes to behavior [27], [28] will require us to look much more closely, and quantitatively, at the behavior of individual organisms.

## Methods

The image centroid and worm shape data were collected as in Ref [13], using tracking video microscopy with sampling frequency 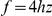, similar to other machine vision-based phenotyping [29], [30]. The resulting centroid time series 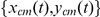 was filtered with a third-order polynomial in a running window spanning 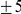 frames and the derivatives used to construct 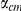 and 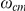 were built from the filtered data. We used a total of 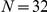 wild-type worms, 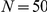 *dop-2 (vs105)* worms and 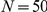 *dop-3 (vs106)* worms, each tracked for 35 minutes. The worms were transferred to the agar plate using a platinum worm pick and we excluded the first 400 seconds of each tracking run to avoid any influences due to mechanical stimulation. Following [13] we derive the shape from each worm image by passing a curve through the center of the body. 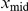 is defined as the center position along this curve. We normalize the arc length 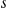 along this curve 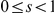, and we define the tangent 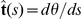. We then rotate all images so that 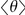 is zero and thus 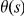 provides a description of the shape that is intrinsic to the worm itself. Finally, we decompose each shape 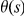 into contributions (modes, 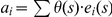) from the leading four eigenworms 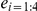, which capture 95% of the variance in shape space. For the linear models connecting modes to movement, Eqn's (5,9), fitting was confined to inter-turn intervals, and the weights were obtained by minimizing the rms error 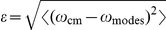.

## Supporting Information

Figure S1
**Reconstructed trajectories.** To explicitly demonstrate that the shape modes provide sufficient information to reconstruct the worm trajectories in real space, we show 16 trajectory reconstructions from (randomly chosen) continuous intervals in the data that are longer than 60 seconds. In these reconstructions, red trajectories are generated from the mode model (Eq 9) while blue trajectories are measured from centroid motion. Arrows denote the direction of motion and the black scale bar is 1 mm in each plot. For these reconstructions, we model the worms trajectory orientation dynamics entirely through 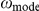 and we use the centroid speed to fit the overall scale. Thus we have 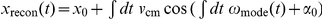 and 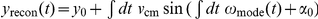. We note that our model is based on 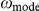 and thus the reconstructions involve two integrations and we match the initial position and initial orientation angle to the worm data. Importantly, during the integration process, errors in the model, however small, accumulate leading to deviations from the actual trajectories at late times. Nonetheless, it is clear from the reconstructions that the shape model predicts qualitatively similar movements even during trajectory epochs where the orientation changes dramatically(0.81 MB EPS)
